# A Comparative Field Study of Indoor Environment Quality and Work Productivity between Job Types in a Research Institute in Korea

**DOI:** 10.3390/ijerph192114332

**Published:** 2022-11-02

**Authors:** Gyu-Bae Lee, Seung-Min Lee, Seung-Eon Lee, Jae-Weon Jeong, Jong-Won Lee

**Affiliations:** 1 Department of Architectural Engineering, College of Engineering, Hanyang University, Seoul 04763, Korea; 2 Korea Institute of Civil Engineering and Building Technology, 283 Goyang-daero, Daehwa-dong, Ilsanseo-gu, Goyang-si 10223, Korea

**Keywords:** indoor environmental quality (IEQ), occupant satisfaction survey, work productivity, research institute, job types

## Abstract

Indoor environment quality (IEQ) evaluation can help improve building satisfaction and productivity of residents. However, for more efficient analysis, it is necessary to gain a large amount of data on the differences between specific groups, such as building and resident work types. In this study, we conducted an IEQ evaluation for administrators and researchers, which are occupational groups of a research institute. The evaluation was conducted using quantitative and qualitative methods to find the relationships between IEQ satisfaction and work productivity for each job type. Our results showed that light environment and office layout were correlated with the work productivity of administrators, and light environment, office layout, thermal comfort, and sound environment were correlated with the work productivity of researchers. In addition, there was a significant difference in layout and thermal comfort items between administrators and researchers. Therefore, this study revealed significant differences in the effect of IEQ evaluation on work productivity between different occupations in a research institute.

## 1. Introduction

Human efforts to build safe and comfortable residential areas have led to the creation of different types of complex buildings and housing styles in modern times. Although the forms of buildings have diversified, people in modern society spend most of their time inside buildings [1]. Thus, indoor environment quality (IEQ) is an important factor in determining and improving the health and satisfaction of the occupants [2,3,4,5]. Moreover, there is a close relationship between IEQ and the productivity of occupants [6,7,8,9]. Previous studies reported that labor cost accounts for 80% of the total operation cost of an organization; therefore, the improvement of IEQ and worker productivity is an important operational issue [10,11,12]. Other studies have mentioned that offices with improved indoor environment can increase the productivity of occupants by more than 20%, which corresponds to more than GBP 130 billion per year [13,14]. In addition, many studies have argued that improved indoor environment can reduce turnover rate and absenteeism by improving occupants’ health and quality of life, thereby enhancing productivity and satisfaction [3,15,16,17,18]. All these cases indicate that IEQ has a significant impact on the building satisfaction, productivity, and health of occupants.

The correlation between IEQ and the satisfaction and productivity of occupants may vary depending on various factors, such as building use, office type, population density, and occupational characteristics. Therefore, in previous studies, building elements, including occupants, were grouped for correlation analysis to elucidate the relationship between occupants and indoor environment. For example, a previous study has shown that women were more likely to be dissatisfied with IEQ items than men [19]. In summers, occupants aged 40 years and older are more satisfied with thermal comfort than those aged less than 40 years [20]. Among the IEQ items, acoustic environment may have a greater impact on occupants with low productivity than on those with high productivity [21]. Occupants whose personal workspaces were far from windows showed generally high satisfaction with most of the IEQ items [22]. It was confirmed that occupants who use a desktop in their workspace tend to prefer lighting compared to those who do not use a desktop [23]. A study conducted in Romania showed that occupants in rural and urban schools may have relatively different perceptions of IEQ owing to such reasons as habits, outdoor environment, and class [24]. Additionally, it was revealed that the correlation between occupants’ satisfaction with buildings and IEQ is related to the size, appearance, and workspace of the building [5]. Furthermore, the correlation between residents and IEQ items was also analyzed for various building types such as airport terminals, libraries, hospitals, and offices [25,26,27,28,29].

The correlation analysis between IEQ satisfaction and occupant productivity has also been studied according to buildings and job type. For example, in higher education institutions, IEQ evaluation was performed mainly for students, and in offices in commercial buildings, IEQ was evaluated by classifying the work types of occupants into “Administrative”, “Technical”, “Professional”, and “Managerial” [30,31,32,33,34]. There are also studies that specifically divided the occupations of inhabitants and analyzed the correlation between their IEQ satisfaction and productivity. Sadick et al. [35] conducted research on the IEQ satisfaction and productivity of students and professors in a university building in a tropical African climate. They found that IEQ had a positive impact on productivity in both groups. Kim et al. [19] found that technical and managerial groups were more dissatisfied with air quality and the amount of lighting than other occupational groups among women working in office buildings. Zuo and MaloneBeach [36] investigated IEQ related to the satisfaction and productivity of workers in assisted living facilities. They found that the non-medical group, composed of managers and administrative staff, was more satisfied with the air quality than the medical group composed of nurses and caregivers. Kamaruzzaman [37] analyzed the correlation between IEQ and the satisfaction of occupants of office buildings and found that the “managerial staff” had the highest average IEQ score among all occupational groups, which included the “clerical and secretarial”, “professional”, “managerial”, and “other” staff. Albuainain [38] investigated the level of IEQ satisfaction for government buildings in Bahrain and analyzed its correlations with non-IEQ factors, such as occupational groups. They found that the IEQ satisfaction level was different depending on the occupational group, and that the technical group had the highest IEQ satisfaction among the five occupational groups.

The results of previous studies can be summarized as follows. IEQ items are important for the work productivity and satisfaction of occupants. In addition, occupants’ evaluation and preference rankings for IEQ items may vary depending on individual factors (e.g., sex and age) or building type and characteristics. In particular, the correlation between job types and IEQ is expected to be diverse, but different research results can be obtained depending on the building and region because related studies are insufficient. Therefore, research on IEQ must continue for various targets and factors, and more correlation data need to be analyzed by constantly studying related scenarios. However, occupants’ evaluation and preference rankings for IEQ items may vary depending on individual factors (e.g., sex and age) or building type and characteristics. Therefore, an increasing number of previous studies are analyzing the IEQ evaluation of residents through correlation with various factors. In previous studies, it was confirmed that the job type was also correlated with the IEQ evaluation of the occupant [30,31,32,33,34,35,36,37,38]. However, because IEQ can obtain different research results depending on buildings and regions, research on various targets and factors should continue, and it is necessary to obtain additional relevant scenarios to analyze correlation data.

As one of the building types, research buildings are also significantly influenced by IEQ in the research efficiency and work productivity of offices [21]. In general, previous studies selected research offices of universities and research offices of commercial companies as targets for the IEQ evaluation of research buildings [21,29]. In addition, the majority of papers analyzing research buildings such as university laboratories focused on analyzing the evaluation of student groups, and did not evaluate employees working in the building [30,31,39,40]. Therefore, it is necessary to analyze IEQ data for non-universal research building and worker types to expand the IEQ evaluation scenario. In this study, the correlation between IEQ items of the public research building in Korea was analyzed. It is meaningful in analyzing occupant’s satisfaction and productivity by job group in a public research building that has been rarely conducted in previous studies. The correlation by the job group studied is expected to contribute to shaping the direction of IEQ improvement in research institution buildings in the future.

The specific objectives of this study were as follows. The first objective was to identify IEQ items that affect building satisfaction and work productivity for each occupational group in the research institute. To this end, quantitative measurements, based on sensors and qualitative assessments based on a survey, were performed. The second objective was to analyze differences in the perception of IEQ between occupational groups in the research institute by comparing the results of quantitative and qualitative evaluations. The statistical significance of each result was analyzed. To this end, an additional literature review was performed.

## 2. Methodology

**Figure 1:**
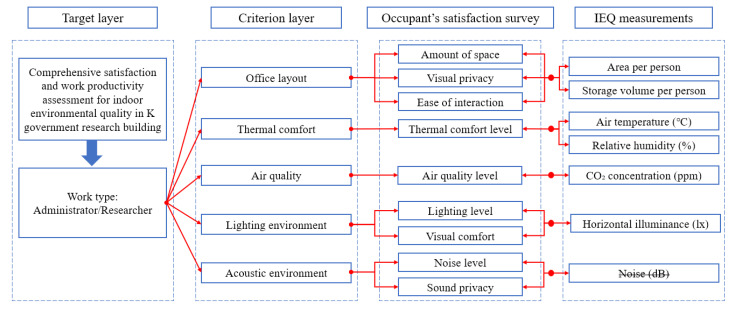
Framework used for analysis in the present study.

The present study focused on measuring IEQ items related to the satisfaction and work productivity of occupants. For evaluating and comparing IEQ items between researchers and administrators, a matching analysis framework was constructed. Figure 1 illustrates the analysis and comparison of quantitative and qualitative data of these two job types in relation to five IEQ items. Each item was based on the IEQ items of the Center for the Built Environment (CBE), in accordance with previous studies [19,21,29,41]. Based on this, the IEQ items measured in this study were classified into five categories: layout, thermal comfort, air quality, lighting environment, and acoustic environment. For the layout item, qualitative data for “amount of space”, “visual privacy”, and “ease of interaction” and quantitative data for “area per person” and “storage volume per person” were compared and analyzed. For the thermal comfort item, qualitative data for “thermal comfort level” and quantitative data for “air temperature” and “relative humidity” were compared and analyzed. For the air quality item, qualitative data for “air quality level” and quantitative data for “CO_2_ concentration” were compared and analyzed. For the lighting environment item, qualitative data for “lighting level” and “visual comfort” and quantitative data for “horizontal illuminance” were compared and analyzed. For the acoustic environment, qualitative data for “noise level” and “sound privacy” were measured and analyzed. The “noise” item, which is a quantitative measure, was not measured in this study.

### 2.1. Occupant Database

**Figure 2:**
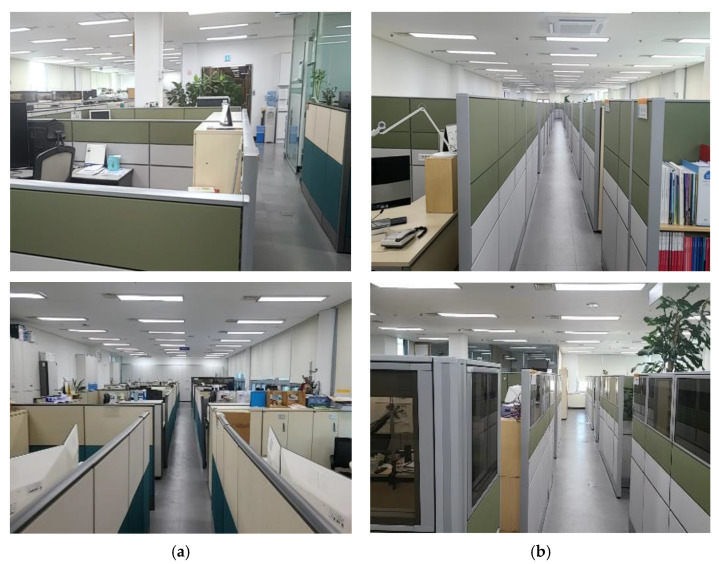
A panoramic view according to the type of office ((**a**) = administrator, (**b**) = researcher).

Table 1 describes the basic information of a total of 98 occupants of the research building K who participated in this study. The data were collected to identify the employees in the institute, and only job type and floor data were used in this study. The data in Table 1 were divided into administrators and researchers, which are the job types within the research building K used in this study. As shown in the table, the administrative group was located on the first (18), second (14), and third (13) floors, whereas the research group was located on the fourth (21) and fifth (32) floors. The numbers in parentheses indicate the number of workers. In both the administrative group and the research group, the proportion of men was higher than that of women, but there was no significant difference. The proportion of people aged 31 to 50 in the administrative group (82.2%) was higher than the research group (67.9%), and the proportion of people aged over 50 in the administrative group (15.6%) was lower than the research group (28.3%). The proportion of workers who worked for three months to one year in the administrative group (46.7%) was higher than that of the research group (35.8%), and the proportion of workers who worked for more than one year (48.9%) was lower than that of the research group (58.5%). In addition, about 90% of the workers in both groups worked more than 30 h per week. Figure 2 shows the panoramic view of the K research building where the research was conducted. The space of the research group was 1.5 times larger than that of the administrative group, resulting in higher density for the administrative group. In addition, 1.2 m cubicles were applied to the administrative group and 1.5 m or larger cubicles for the research group owing to the layout difference. A survey was conducted for approximately 14 days from December 14 to 28, 2021. The offices were equipped with basic facilities with no special facility (one computer per person and a partition height of 1.2–1.5 m).

### 2.2. Physical Measurements

**Figure 3:**
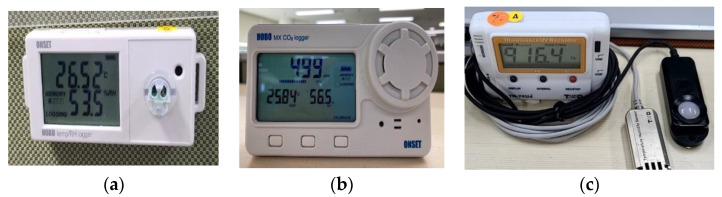
Sensors used in the study ((**a**) = UX100-011A, (**b**) = MX1102A, (**c**) = TR-74Ui-S).

In the case of quantitative measurements, the main IEQ parameters identified in related studies were selected. For thermal comfort, air temperature and relative humidity were measured with consideration for window and interior positions [42,43]. In previous studies, quantitative evaluation of air quality was generally performed through CO_2_ concentration measurement [28,44,45,46]. Therefore, in this study, the air quality item measured the indoor ventilation level and the freshness of air by deriving the CO_2_ concentration from the representative value of each floor. For assessing the lighting environment, a sensor that could measure the brightness of natural light and lighting was installed and operated [47,48,49]. In this study, however, decibel measurement was not performed using a sound level meter. Table 2 and Figure 3 show the accuracy information on the sensors. Each sensor was pre-measured at the same location for 24 h prior to quantitative measurement to ensure that the error range was within the accuracy of each instrument. As a result of the pre-measurement, it was confirmed that each sensor was operating within the error range, so the measured values of each sensor were analyzed for common use.

**Figure 4:**
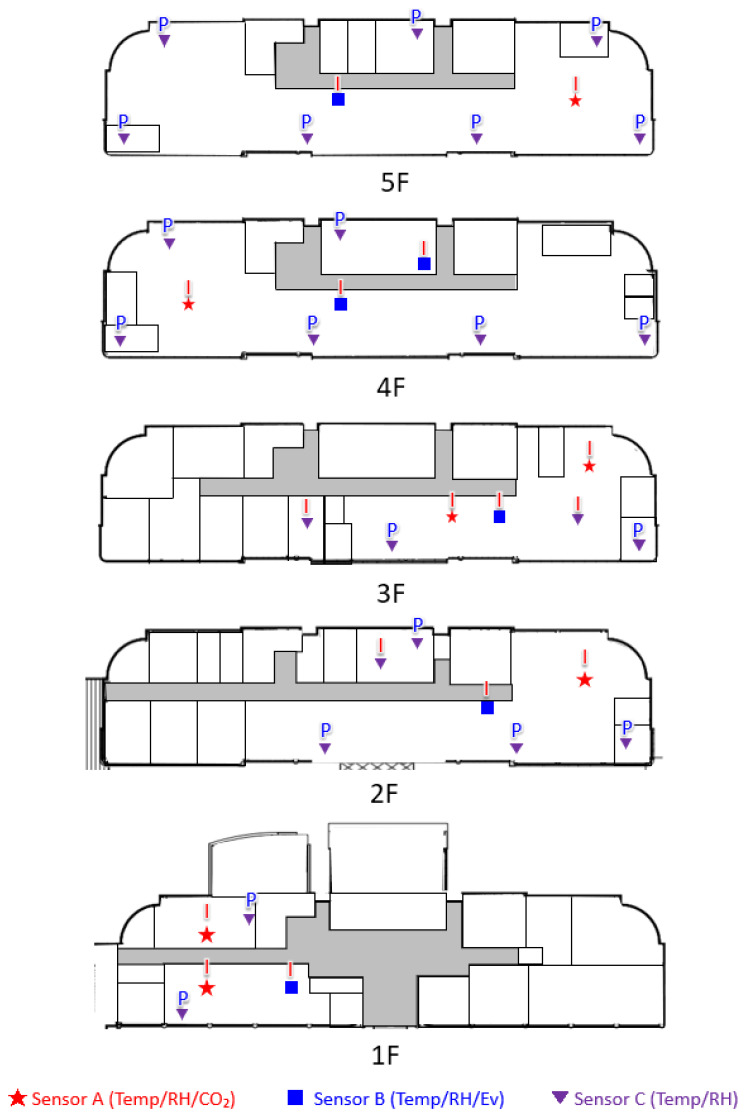
Sectional layout of each floor of the research institute. For security reasons, the floor plan is presented in limited details. The symbol P stands for the period zone, and the symbol I stands for the interior zone. Sensors A, B, and C are the same as those shown in Figure 3.

Figure 4 shows the sensor installation positions. The sensors were positioned so that the representative values of the perimeter zone and the interior zone could be identified in consideration of the number of equipment possessed. The sensors were installed at a height of approximately 1.2 m from the floor to perform continuous measurements, and the data obtained during flexible work hours (07:00–19:00) were analyzed. Most measurements were performed during work hours. The window shades could be adjusted individually, and personal computers and the heating system were in normal operation. All the sensors performed measurements at the same time during the aforementioned period, and the measurements were recorded every 10 min.

### 2.3. Qualitative Evaluation

In the case of qualitative measurement, based on CBE’s survey data, it was performed through a questionnaire partially revised to suit the purpose of the study and the climate of Korea. In addition, exploratory factor analysis was conducted to examine whether the questionnaire used in the study could reliably identify the factors determining occupant satisfaction in each group. As a result of exploratory factor analysis, the Kaiser–Meyer–Olkin (KMO) measure was 0.641, and the significance probability was less than 0.05 in the Bartlett’s sphericity test results, thereby confirming that the questionnaire was suitable. Moreover, reliability analysis was conducted to examine the internal consistency of each factor. Cronbach’s alpha was used to determine the internal consistency, and the Cronbach α value for each variable ranged from 0.767 to 0.941. This result was satisfactory, as a value of 0.6 or higher was recommended in previous studies to determine whether the questionnaire was appropriate [21,29,50]. After exploratory factor analysis, the questionnaire was distributed and collected during the same period as the quantitative measurement period. A total of 150 questionnaires were returned, out of which 98 were valid (a valid response rate of 65.3%). In these valid responses, 36 questionnaires (14 males and 22 females) were from administrators and 62 questionnaires (45 males and 17 females) from researchers. Therefore, in this study, data for 98 people were qualitatively analyzed. Quantitative evaluation was performed by partially reconstructing according to the climate of Korea by referring to the IEQ evaluation items of CBE [41]. For the evaluation of IEQ in the target building, five items (layout, thermal comfort, air quality, lighting environment, and acoustic environment) were analyzed. In addition, total productivity items were analyzed to evaluate overall work productivity for the overall environmental conditions and to find out how the evaluated work productivity and overall work productivity evaluation in each IEQ item correlated. The survey began with the collection of individual information, such as sex, age, and number of years in the office, from the respondents. The degree of comfort felt by each respondent was then measured for assessing the productivity, five IEQ items, and other factors. Satisfaction evaluation for each item was conducted with a survey reflecting the 7-point Likert scale from 1 (very dissatisfied) to 7 (very satisfied), as studied in CBE and ASHRAE 55 [41,42]. In addition, total productivity and work productivity were evaluated through a survey on the same 7-point Likert scale from 1 (very low) to 7 (very high). In addition to evaluating satisfaction and productivity for each IEQ item, additional options (e.g., causes of dissatisfaction and time zones of dissatisfaction) that may be used in future studies were also collected. Table 3 shows the details of the survey.

## 3. Results and Analysis

### 3.1. Results of Quantitative Measurements

Table 4 shows the comparison of air temperature, relative humidity, horizontal illuminance, CO_2_ concentration, area per person, and storage volume per person between administrators and researchers, as analyzed using the Mann–Whitney U-test. The results can be summarized for each IEQ item as follows:

(1) For the office layout, there was a significant difference between the research and administrative groups. Each item is a parameter closely related to the personal space of occupants in the office. Area per person was calculated using the formula “room area/seat number”, whereas storage volume per person was determined using the formula “total volume of cabinets/seat number” [29]. For the office layout, the difference in area per person was not significant, but storage volume per person exhibited a significant difference (*p* < 0.001). In the target building, the average area per person and storage volume per person of the research group were 16.9 m^2^ and 1.4 m^3^, respectively, which were relatively higher than those of the administrative group (14.8 m^2^ and 0.8 m^3^, respectively). This finding indicates that the average personal space of occupants in the research group was considerably larger than that in the administrative group.
(2) For the thermal environment, there was no significant difference in indoor humidity between administrators and researchers. However, indoor temperature was significantly different between administrators and researchers (*p* < 0.01). According to EN 16798-1:2019 [51], the recommended temperature range is 19–25 °C, and the recommended humidity range is 25–60% for office buildings. This confirmed that the relative humidity of the target building was lower than the recommended range. For temperature, the recommended range was satisfied in most of the rooms but was not met on the fourth floor, which belonged to the research group. In addition, the average temperature of the research group (25.0 °C) was 1.1 °C higher than that of the administrative group (23.9 °C). Therefore, on average, the physical environment of the administrative group was better than that of the research group.
(3) For the air quality item, there was no significant difference in CO_2_ concentration between the research and administrative groups. According to the recommendation by BS EN standard 15251:2007, CO_2_ concentration in offices must be less than 800 ppm [48]. According to the data, CO_2_ concentration in several sections where the administrative group worked exceeded the recommended range. In addition, the average CO_2_ concentrations of the research and administrative groups were 586.0 and 676.5 ppm, respectively. This indicates that the research group had a better environment than the administrative group in terms of air quality.
(4) For the lighting environment item, there was no significant difference between the administrative and research groups. The average illuminance of the administrative group (1048.0 lx) was higher than that of the research group (927.8 lx). According to BS EN standard 12464-1:2011, the recommended illuminance for offices is 500 lx or higher [42]. Therefore, the recommended illuminance in the target building was satisfied for both the administrative and research groups. However, the lighting intensity of the administrative group was higher than that of the research group.
(5) Acoustic environment was not included in this study.

### 3.2. Results of Qualitative Evaluation

#### 3.2.1. Evaluation of IEQ Aspects and their Impacts on Work Productivity

Table 5 shows the results of the qualitative assessment. The average satisfaction of the occupants with IEQ is presented in the table. For the administrative group, lighting environment (5.10) showed the highest satisfaction, followed by thermal comfort (4.71), layout (4.52), air quality (4.02), and acoustic environment (3.60). For the research group, layout (5.15) exhibited the highest satisfaction, followed by lighting environment (5.11), air quality (3.97), thermal comfort (3.77), and acoustic environment (3.58). Both groups showed high satisfaction with lighting environment, and there was a difference in the average satisfaction with layout and thermal comfort. In addition, significant differences in layout, thermal comfort, air quality, lighting environment, and acoustic environment between the administrative and researcher groups were examined. As the data did not pass the normality test, the Mann–Whitney test was applied for data analysis. Table 6 shows the comparison of the average satisfaction with the target building, as analyzed using the Mann–Whitney test. The satisfaction data for each item in Table 5 was used for the analysis, and the symbol M in Table 6 means the average ranking value of IEQ items by job type. In Table 6, when the *p*-value of the corresponding IEQ item is significant, the higher the average ranking value, the higher the average satisfaction with the item. As a result of the Mann–Whitney test, there were significant differences in layout (Z = −2.757, *p* < 0.01) and thermal comfort (Z = −2.808, *p* < 0.01) between the occupational groups. In the target building, the average ranking of researchers (M = 55.48) was higher than that of administrators (M = 39.21) for layout, and the average ranking of administrators (M = 59.90) was higher than that of researchers (M = 43.46) for thermal comfort. In contrast, there was no significant difference in air quality, lighting environment, and acoustic environment (*p* > 0.05).

Table 7 shows the work productivity evaluation of the occupants by IEQ item, and shows the total productivity evaluation for the entire work environment. Significant differences in total productivity, layout productivity, thermal comfort productivity, air quality productivity, lighting environment productivity, and acoustic environment productivity were determined between the administrative and research groups. As the data did not pass the normality test, the Mann–Whitney test was applied. As shown in Table 8, there were significant differences in layout productivity (Z = −2.136, *p* < 0.05, Mann–Whitney test) and thermal comfort productivity (Z = −2.252, *p* < 0.05, Mann–Whitney test) between the occupational groups. In the target building, the average ranking of researchers (M = 54.01) was higher than that of administrators (M = 41.74) for layout productivity, and the average ranking of administrators (M = 57.83) was higher than that of researchers (M = 44.66) for thermal comfort productivity. On the contrary, there was no significant difference in total productivity, air quality productivity, lighting environment productivity, and acoustic environment productivity (*p* > 0.05).

Spearman correlation analysis was conducted to examine the correlation of total productivity with layout, thermal environment, air quality, lighting environment, and acoustic environment, and the results are presented in Table 9. For administrators, lighting environment (0.444) showed the largest absolute coefficient value, followed by layout (0.370), air quality (0.326), acoustic environment (0.319), and thermal comfort (0.251). For researchers, layout (0.507) exhibited the largest absolute value, followed by lighting environment (0.387), thermal comfort (0.325), acoustic environment (0.319), and air quality (0.160). In addition, the work productivity of administrators showed a significant positive correlation with layout (r = 0.370, *p* < 0.05) and lighting environment (r = 0.444, *p* < 0.01), whereas the work productivity of researchers exhibited a significant positive correlation with layout (r = 0.507, *p* < 0.001), thermal environment (r = 0.325, *p* < 0.05), lighting environment (r = 0.387, *p* < 0.01), and acoustic environment (r = 0.319, *p* < 0.05).

#### 3.2.2. Evaluation of Sub-Factors of IEQ Aspects

The Mann–Whitney test was conducted to determine differences in the sub-factors of layout, thermal comfort, air quality, lighting environment, and acoustic environment between the administrative and research groups. For administrators, thermal comfort (59.90) showed the highest average ranking value, followed by sound privacy (50.24), lighting (47.42), ease of interaction (47.25), noise level (45.97), visual comfort (44.15), air quality (43.11), visual privacy (40.46), and the amount of space (39.79), as shown in Table 10. For researchers, the amount of space (55.14) exhibited the highest average ranking value, followed by visual privacy (54.75), air quality (53.21), visual comfort (52.60), noise level (51.55), ease of interaction (50.81), lighting (50.71), sound privacy (49.07), and thermal comfort (43.46). In addition, there were significant differences in the amount of space (Z = −2.658, *p* < 0.01), visual privacy (Z = −2.443, *p* < 0.05), and thermal comfort (Z = −2.808, *p* < 0.01), which are the sub-factors of layout, between the occupational groups. The average ranking of researchers (M = 55.14) was higher than that of administrators (M = 39.79) for the amount of space. The average ranking of researchers (M = 54.75) was higher than that of administrators (M = 40.46) for visual privacy, and the average ranking of administrators (M = 59.90) was higher than that of researchers (M = 43.46) for thermal comfort. On the contrary, there was no significant difference in ease of interaction, which is the sub-factor of layout, as well as air quality, lighting environment, and acoustic environment (*p* > 0.05).

## 4. Discussion

Table 11 summarizes the differences in the quantitative and qualitative evaluation results for key IEQ aspects between the administrative and research groups.

In the case of layout, as a result of the quantitative measurement shown in Table 4, the difference in area per capita between the group of researchers and the group of administrators was not statistically significant, but the difference in storage per capita was significant (*p* < 0.001). In addition, in the qualitative evaluation in Table 6, the administrative group (39.21) had lower average satisfaction with the placement than the research group (55.48), and this difference was statistically significant (*p* < 0.01, Mann–Whitney test). Moreover, in Table 8, the manager group (41.74) had a statistically significantly lower productivity evaluation for batches than the research group (54.01) (*p* < 0.05, Mann–Whitney test). These results are basically inferred because the research group received more office supplies due to the larger office space and higher partitions than the manager group. Similarly, previous studies have pointed out that problems can occur when offices are dense or have small storage space [21,52]. Therefore, through this study, it was confirmed that layout can have a great influence on the satisfaction and work productivity of residents in laboratory buildings.

In the case of thermal comfort, the temperature and humidity of the work space were quantitatively measured. According to EN 16798-1:2019 [51], it is recommended that the average temperature in winter workspaces be maintained in the range of 19 to 25 °C and humidity in the range of 25 to 60%. In Table 4, in the case of humidity, neither group belonged to the recommended range, but it was not a statistically significant result. However, in the case of temperature, the average temperature of the research group (25.0 °C) was found to be higher than the average temperature of the administrative group (23.9 °C), and it was found to be statistically significant (Table 4). In the qualitative evaluation of Table 6, the administrative group (59.90) had a higher average ranking value in terms of satisfaction than the research group (43.46), and this difference was significant (*p* < 0.01, Mann–Whitney test). Similarly, in Table 8, which evaluated work productivity, the administrative group (57.83) had significantly higher productivity evaluation for thermal comfort than the research group (44.66) (*p* < 0.05, Mann–Whitney test). As shown in Table 11, in quantitative and qualitative evaluations, the research group with high average temperature had the same lower satisfaction and productivity than the manager group, and it was statistically significant. This is consistent with previous studies showing that building temperatures affect building satisfaction and work productivity of residents [53,54,55,56]. Therefore, even in laboratory buildings, thermal comfort was found to have a significant association between employee satisfaction and work productivity. In the case of air quality, both the quantitative and qualitative results showed no significant difference between the research and administrative groups. In the quantitative measurement results, CO2 concentration met the recommended range (less than 800 ppm) presented by BS EN standard 15251:2007 [48] for both researchers and administrators. As a result of the qualitative evaluation, it was confirmed that there was no statistically significant difference in both satisfaction and work productivity in the two groups (Table 6 and Table 8). In addition, there was no statistically significant correlation between the total productivity and work productivity of IEQ items (Table 9). Therefore, in this study, it can be concluded that air quality is not a factor that greatly affects satisfaction and productivity for both administrators and researchers of the K research building. However, this is a result limited to the K research building where the study was conducted, and the data are insufficient to generalize. Therefore, it is believed that research on the IEQ evaluation of the research building should be continuously conducted. For the lighting environment, the quantitative measurement results exceeded 500 lx, conforming to BSEN standard 12464-1:2011 [47], and the average illuminance of the manager group (1149.1 lx) was higher than that of the research group (932.8 lx). As a result of qualitative evaluation, there was no statistically significant difference in the lighting environment between the two groups in terms of satisfaction and work productivity. In previous studies, it was mentioned that the lighting environment had a significant association with the IEQ of occupants of the laboratory building [21,29]. Other previous studies have noted that satisfaction with the lighting environment is associated with the health and productivity of the occupants [57,58]. Furthermore, in the Spearman test in Table 9, the lighting environment was found to be statistically significant in the association between total and work productivity in both groups. Therefore, the association analysis of IEQ with the lighting environment of the research building conducted in this study should supplement the data with additional survey data, and further research on the association with non-occupational factors or environmental factors is needed.

In the case of the acoustic environment, quantitative evaluation was not performed due to the security of the government laboratory building, but only qualitative evaluation was performed. In Table 5 and Table 7, which are the results of the survey, the satisfaction and productivity of the sound environment were very low compared to other IEQ items. In some previous studies, the IEQ evaluation for the acoustic environment was also confirmed to be the lowest [19,21]. Previous studies have shown that every day and various noise sources such as conversations, phone ringtones, and computer typing have a significantly negative relationship with satisfaction with the acoustic environment in the office [56]. In addition, due to the features of the research building, the requirements for the sound environment may be high because of the complex and concentration-dependent work being carried out [29]. However, in the Mann–Whitney test, which determines whether the results of the survey conducted in this study are statistically significant, the results in Table 6 and Table 8 prove that the survey values for the acoustic environment conducted in this study were not significant. Although Table 9 shows that total productivity and researchers’ work productivity are statistically significant, comprehensively considered, it is considered that additional factors need to be correlated to evaluate the sound environment of residents in research buildings.

## 5. Conclusions

In this study, the IEQ satisfaction and productivity of administrators and researchers in the research building K in Korea were evaluated and compared using quantitative measurements and a qualitative survey, respectively. The main results can be summarized as follows:

(1) The IEQ items with statistically significant differences between the research group and the administrative group in the research building were the layout and thermal comfort. The two items showed significant differences in both quantitative and qualitative evaluations. In addition, in the total productivity item that evaluated the relationship between overall work environment and work productivity, the administrative group was associated with lighting environment and layout items, and the research group was associated with layout, lighting environment, thermal comfort, and sound environment.
(2) Among the surveyed IEQ items, the statistically significant layout and thermal comfort were consistent with the results of quantitative and qualitative analysis. In the quantitative measurement results for the target building, the layout environment was more positive for researchers than for administrators, and the thermal comfort was more positive for administrators than for researchers. In addition, the qualitative data showed that researchers were more satisfied with the layout than administrators, while administrators were more satisfied with the thermal comfort than researchers. This echoes the results of previous studies that the more comfortable the space arrangement and thermal comfort are, the better the satisfaction and work productivity of the residents. Therefore, as a result of this study, if the difference between IEQ items between groups is statistically significant, it is inferred that there is a possibility that the quantitative and qualitative evaluation results may be the same.

However, this study has the following limitations. Since this study was conducted on specific buildings in a specific climate, there is insufficient data to conclude with an IEQ evaluation representing the research building. In addition, since the IEQ evaluation of this study is concentrated on the research and administrative groups, a study that considers additional indicators or environmental factors should be conducted. Further research should also be conducted on the acoustic environment because no quantitative measurements have been made for security reasons in the building. Therefore, it is believed that the results of this study need to be improved through continuous investigation and data update. Nevertheless, this study was conducted on research buildings of government agencies that were rarely used in IEQ evaluation of existing research buildings, and significant comparison results were obtained for the laboratory occupational group. Therefore, the results of this study are expected to be useful for IEQ evaluation for various occupational groups of research institutes and similar buildings. In the future, IEQ evaluation will be conducted with various building groups, individual elements, and environments. Through this, it is expected that it will be possible to establish a research method that provides optimal building IEQ to residents and increases work productivity.

## Figures and Tables

**Table 1:** Basic information on the administrator and researcher floors.

Type		Administrator					Researcher			
Floor		1F	2F	3F	Total (N)	Total (%)	4F	5F	Total (N)	Total (%)
Number of occupants who responded to survey		18	14	13	45	100.0	21	32	53	100.0
Sex	Male	8	6	9	23	51.1	16	13	29	54.7
	Female	10	8	4	22	48.9	5	19	24	45.3
Age	Under 30	0	1	0	1	2.2	2	0	2	3.8
	31 to under 50	15	11	11	37	82.2	13	23	36	67.9
	Over 50	3	2	2	7	15.6	6	9	15	28.3
Working experience	Under 3 months	1	0	1	2	4.4	1	2	3	5.7
	More than 3 months and less than 1 year	10	6	5	21	46.7	13	6	19	35.8
	Over 1 year	7	8	7	22	48.9	7	24	31	58.5
Weekly working hours	Under 10 h	2	0	0	2	4.4	1	4	5	9.4
	11 h to under 30 h	0	1	1	2	4.4	1	0	1	1.9
	Over 30 h	16	13	12	41	91.1	19	28	47	88.7

**Table 2:** Information on measurement equipment.

Equipment	Measurement Parameter	Accuracy	Measurement Time
Data Logger (UX100-011A)	Air temperature	±0.21 °C from 0 to 50 °C	10 min (at each point)
	Relative humidity	±2.5% from 10% to 90%	
Data Logger (MX1102A)	Air temperature	±0.21 °C from 0 to 50 °C	
	Relative humidity	±2% from 20% to 80%	
	CO_2_ concentration	±50 ppm ±5% reading at 25 °C, less than 90% RH	
USB Connectable Loggers (TR-74Ui-S)	Air temperature	±0.3 °C at 10 to 40 °C	
	Relative humidity	±2.5% at 15 to 35 °C, 30% to 80%	
	Illuminance	±5% at 25 °C, 50% RH	

**Table 3:** Information of IEQ survey items.

Items	Questionnaire Items	Survey Questions
Layout	Amount of space	How satisfied are you with the amount of space in your workspace?
		How improved is your productivity with the amount of space in your workspace?
	Visual privacy	How satisfied are you with the visual privacy in your workspace?
		How improved is your productivity with visual privacy in your workspace?
	Ease of interaction	How satisfied are you with the ease of interaction in your workspace?
		How improved is your productivity with the ease of interaction in your workspace?
Thermal productivity	Thermal comfort level	How satisfied are you with the thermal comfort level in your workspace?
		How improved is your productivity with the thermal comfort level in your workspace?
Air quality	Air quality level	How satisfied are you with the air quality level in your workspace?
		How improved is your productivity with the air quality level in your workspace?
Lighting environment	Lighting level	How satisfied are you with the lighting level in your workspace?
		How improved is your productivity with the lighting level in your workspace?
	Visual comfort	How satisfied are you with the visual comfort in your workspace?
		How improved is your productivity with the visual comfort in your workspace?
Acoustic environment	Noise level	How satisfied are you with the noise level in your workspace?
		How improved is your productivity with the noise level in your workspace?
	Sound privacy	How satisfied are you with the sound privacy in your workspace?
		How improved is your productivity with the sound privacy in your workspace?
Total productivity	Perceived work productivity level	How much has overall work productivity improved through the overall environmental conditions of the workspace?

**Table 4:** Comparison of quantitative measurements between the administrative and research groups, analyzed using the Mann–Whitney U test.

Classification	Administrator				Researcher			Z ^b^	*p*-Value ^c^	R ^d^-Value
	1F ^a^	2F	3F	Mean	4F	5F	Mean			
Temperature (°C)	23.7	23.3	24.8	23.9	25.7	24.2	25.0	−2.681	0.007 *	19–25 [51]
Humidity (%)	18.4	19.5	18.5	18.8	17.0	18.2	17.6	−1.566	0.117	25–60 [51]
Ev (lx)	927.0	1035.0	1182.0	1048.0	806.5	1049.0	927.8	−0.655	0.513	≥500 [47]
CO_2_ (ppm)	645.0	750.0	634.5	676.5	594.0	578.0	586.0	−1.549	0.121	≤800 [48]
APP (m^2^)	12.8	14.0	17.6	14.8	18.3	15.5	16.9	−0.577	0.564	-
SVPP (m^3^)	0.8				1.4			−6.403	0.000 **	-

^a^ Floor; ^b^ Z distribution; ^c^ analyzed using the Mann–Whitney U test (* *p*-value <0.01, ** *p*-value < 0.001); ^d^ recommended; Ev, horizontal illuminance; APP, area per person; SVPP, storage volume per person.

**Table 5:** Respondents’ perception of satisfaction with indoor environment quality (IEQ) items (Likert 7-point scale).

	Administrator	Researcher
Layout	4.52	5.15
Thermal comfort	4.71	3.77
Air quality	4.02	3.97
Lighting environment	5.10	5.11
Acoustic environment	3.60	3.58

**Table 6:** Qualitative assessment of satisfaction in administrators and researchers, analyzed using the Mann–Whitney U test.

	M ^a^ (Administrator)	M (Researcher)	U ^b^	Z	*p*-Value ^c^
Layout	39.21	55.48	745.500	−2.757	0.006 *
Thermal comfort	59.90	43.46	741.500	−2.808	0.005 *
Air quality	43.11	53.21	886.000	−1.736	0.083
Lighting environment	45.50	51.82	972.000	−1.091	0.275
Acoustic environment	48.08	50.32	1065.000	−0.379	0.705

^a^ M average ranking value; ^b^ Mann–Whitney U; ^c^ * *p*-value < 0.01.

**Table 7:** Respondents’ perception of productivity in relation to IEQ aspects (Likert 7-point scale).

	Administrator	Researcher
Total productivity	4.79	5.08
Layout	3.99	4.62
Thermal comfort	4.58	3.89
Air quality	4.14	3.91
Lighting environment	4.87	4.71
Acoustic environment	3.71	3.73

**Table 8:** Qualitative assessment of productivity in administrators and researchers, analyzed using the Mann–Whitney U test.

	M (Administrator)	M (Researcher)	U	Z	*p*-Value ^a^
Total productivity	45.29	51.94	964.500	−1.147	0.251
Layout	41.74	54.01	836.500	−2.136	0.033 *
Thermal comfort	57.83	44.66	816.000	−2.252	0.024 *
Air quality	46.67	51.15	1014.000	−0.770	0.441
Lighting environment	45.19	52.00	961.000	−1.193	0.233
Acoustic environment	51.81	48.16	1033.000	−0.623	0.533

^a^ * *p*-value < 0.05.

**Table 9:** Spearman rank correlation coefficients of work productivity and IEQ aspects.

	Layout	Thermal Comfort	Air Quality	Lighting Environment	Acoustic Environment
Productivity (Administrator)	0.370 *	0.251	0.326	0.444 **	0.319
Productivity (Researcher)	0.507 ***	0.325 *	0.160	0.387 **	0.319 *

* *p*-value < 0.05. ** *p*-value < 0.01, *** *p*-value < 0.001.

**Table 10:** Comparison of average satisfaction with sub-factors.

	Items	M (Administrator)	M (Researcher)	U	Z	*p*-Value ^a^
Layout	Amount of space	39.79	55.14	766.500	−2.658	0.008 **
	Visual privacy	40.46	54.75	790.500	−2.443	0.015 *
	Ease of interaction	47.25	50.81	1035.000	−0.625	0.532
Thermal comfort	Thermal comfort	59.90	43.46	741.500	−2.808	0.005 **
Air quality	Air quality	43.11	53.21	886.000	−1.736	0.083
Lighting environment	Lighting	47.42	50.71	1041.000	−0.581	0.561
	Visual comfort	44.15	52.60	923.500	−1.482	0.138
Acoustic environment	Noise level	45.97	51.55	989.000	−0.952	0.341
	Sound privacy	50.24	49.07	1089.500	−0.199	0.842

^a^ * *p*-value < 0.05. ** *p*-value < 0.01.

**Table 11:** Differences in quantitative and qualitative assessment results between the administrative and research groups.

Key IEQ Aspects	Evaluation Results Based on Quantitative Measurements	Evaluation Results Based on Qualitative Evaluation	Evaluation Results Based on Qualitative Assessment of Productivity	Is Qualitative Evaluation Consistent with Quantitative Results?
Layout	Researchers better than Administrators	Researchers better than Administrators	Researchers better than Administrators	YES
Thermal comfort	Administrators better than Researchers (Temperature)	Administrators better than Researchers	Administrators better than Researchers	YES

Note: Only items with significant differences between the administrative and research groups are included in this table. The humidity parameter does not meet this requirement.

## Data Availability

The data presented in this study are available on request from the corresponding author. The data are not publicly available due to ethical reasons.
